# Association between Interleukin-6 Levels and Perioperative Fatigue in Gastric Adenocarcinoma Patients

**DOI:** 10.3390/jcm8040543

**Published:** 2019-04-20

**Authors:** Jin-Ming Wu, Hui-Ting Yang, Te-Wei Ho, Shiow-Ching Shun, Ming-Tsan Lin

**Affiliations:** 1 Department of Surgery, National Taiwan University Hospital and College of Medicine, National Taiwan University, 7 Chung-Shan South Rd. Taipei 10002, Taiwan; wujm0531@ntu.edu.tw (J.-M.W.); skbaskba@gmail.com (T.-W.H.); 2 School of Nursing, College of Medicine, National Taiwan University, Taipei 10002, Taiwan; sophy790303@gmail.com (H.-T.Y.); scshun@ntu.edu.tw (S.-C.S.)

**Keywords:** cancer fatigue, cytokines, gastrectomy, gastric cancer, cachexia

## Abstract

Background: Gastric adenocarcinoma (GA), one of the most common gastrointestinal cancers worldwide, is often accompanied by cancer cachexia in the advanced stage owing to malnutrition and cancer-related symptoms. Although resection is the most effective curative procedure for GA patients, it may cause perioperative fatigue, worsening the extent of cancer cachexia. Although the relationship between cytokines and cancer fatigue has been evaluated, it is unclear which cytokines are associated with fatigue in GA patients. Therefore, this study aimed to investigate whether the changes in cytokine levels were associated with the perioperative changes in fatigue amongst GA patients. Methods: We included GA patients undergoing gastric surgery in a single academic medical center between June 2017 and December 2018. Fatigue-related questionnaires, serum cytokine levels (interferon-gamma, interleukin (IL)-1, IL-2, IL-5, IL-6, IL-12 p70, tumor necrosis factor-alpha, and granulocyte-macrophage colony-stimulating factor), and biochemistry profiles (albumin, prealbumin, C-reactive protein, and white blood cell counts) were assessed at three time points (preoperative day 0 (POD 0), post-operative day 1 (POD 1), and postoperative day 7 (POD 7)). We used the Brief Fatigue Inventory-Taiwan Form to assess the extent of fatigue. The change in fatigue scores among the three time points, as an independent variable, was adjusted for clinicopathologic characteristics, malnutrition risk, and cancer stages. Results: A total of 34 patients were included for analysis, including 12 female and 22 male patients. The mean age was 68.9 years. The mean score for fatigue on POD 0, POD 1, and POD 7 was 1.7, 6.2, and 3.6, respectively, with significant differences among the three time points (*P* < 0.001). Among the cytokines, only IL-6 was significantly elevated from POD 0 to POD 1. In the regression model, the change in IL-6 levels between POD 0 and POD 1 (coefficients = 0.01 for every 1 pg/mL increment; 95% confidence interval: 0.01–0.02; *P* = 0.037) and high malnutrition risk (coefficients = 2.80; 95% confidence interval: 1.45–3.52; *P* = 0.041) were significantly associated with changes in fatigue scores. Conclusions: The perioperative changes in plasma IL-6 levels are positively associated with changes in the fatigue scores of GA patients undergoing gastric surgery. Targeting the IL-6 signaling cascade or new fatigue-targeting medications may attenuate perioperative fatigue, and further clinical studies should be designed to validate this hypothesis.

## 1. Introduction

Worldwide, gastric adenocarcinoma (GA) is among the most common causes of cancer mortality—GA ranks fifth in terms of the incidence (approximately 952,000 cases in 2012) and third in terms of mortality (approximately 723,000 cases in 2012) [1]. In Taiwan, gastric cancer was the fifth leading cause of cancer-related deaths in 2012. Notably, GA patients often had poor physical conditions before surgery because of cancer-related complications, such as anemia (due to bleeding caused by cancer), malnutrition, cancer cachexia, or gastrointestinal discomfort due to obstructions of the gastrointestinal tract or cancer bleeding [2,3]. Surgical intervention is the main curative modality for gastric cancer, including removal of part of the stomach depending on the location of cancer and radical dissection of the lymph nodes. However, it has a significantly negative impact on patients because of physical damage and surgical stress, which is a spectrum of changes occurring throughout different systems in the body, including elevated adrenocorticotropic hormone levels, hyperglycemia, immunological changes, and an immunosuppressive effect [4]. Therefore, in extremely ill patients, complex oncological operations have high morbidity and mortality rates. 

Fatigue is one of the most widely reported complaints during perioperative periods and is described as involving tiredness, lethargy, sluggishness, changed emotional state, and a desire to sleep more [5]. Postoperative fatigue is associated with the surgical complexity, while it may occur even after uncomplicated operations [6,7]. Fatigue increased in the first 2 weeks after the operation, was restored to preoperative levels within 1 month, and improved further at 3 months following surgery [8,9]. Despite the prevalence and impact of fatigue following surgery, the etiology of postoperative fatigue remains poorly understood. In addition to surgery-related fatigue, GA patients experience another etiology of fatigue—cancer-related fatigue, defined as a distressing, persistent, subjective sense of emotional, physical, or cognitive tiredness or exhaustion associated with cancer or cancer treatment that is not proportional to recent activity and interferes with usual functioning [10,11]. Cancer cachexia occurs in approximately 80% of cancer patients and accounts for the primary cause of cancer-related death [12,13]. 

The pathogenesis of cancer-related fatigue is multifactorial, and many mechanisms may contribute to its development, including anemia, anti-cancer treatment, hormones, cancer progression, or cytokines [12,13,14,15]. Fatigue is a frequently under-recognized complication in cancer patients until they have lost more than 5–7% of body mass and have a significantly reduced quality of life [16,17]. Therefore, routine screening for fatigue is an important component in improving the quality of life of patients living with cancer.

Fatigue was associated with cytokine dysregulation (interleukin-1, tumor necrosis factor, or glucocorticoids) in patients with breast or prostate cancer [18,19,20,21,22,23]. One recent study showed that acute-phase proteins and cytokines (interleukin-1 and interleukin-6) were elevated in gastrointestinal cancer patients with cachexia or fatigue compared to normal populations [24]. However, anti-cancer treatment interfered with the process of cytokine-medicated fatigue [19], which might have altered the mechanism of cytokines in GA patients undergoing major operations, thereby resulting in physical insults and wound pain. Hence, a study focusing on the perioperative status of fatigue in GA patients is imperative. Therefore, this study aimed to validate the relationship between perioperative fatigue and cytokine levels in GA patients. 

## 2. Methods

Adult GA patients (aged ≥ 20 years) undergoing gastric operations were included in this study between June 2017 and December 2018 at the National Taiwan University Hospital. The exclusion criteria were previous abdominal surgery, inability to communicate verbally and completely fill out the questionnaires, or severe renal or hepatic impairment. 

This study was reviewed and approved by the Research Ethics Committee of the National Taiwan University Hospital (201705112RINB) and followed all ethical recommendations. All participating patients were informed about the study processes and signed the informed consent form. 

### 2.1. Instruments

We used the Brief Fatigue Inventory-Taiwan Form (BFI-T), a scale with 11 points (0 to 10), to assess fatigue [25]. For each question, 0 was considered ‘‘no fatigue’’ and 10 was the ”worst fatigue possible”. Among the questions, three questions measured the severity of fatigue currently, in daily situations, and in the last 24 h; and six questions measured the influence of fatigue on general activities, mood, ability to walk and work, relationship with other people, and recreational activities [26]. The total score was the average score of all the answers, and fatigue was categorized as mild (1 to 3 points), moderate (4 to 6 points), or severe (7 to 10 points) according to the total score. The use of the BFI for gastric cancer patients has already been validated [27,28]. In this study, the BFI-T questionnaire will be filled out on the admission date before surgery (POD 0; baseline status), on postoperative day 1 (POD 1), and on postoperative day 7 (POD 7). To survey the severity of fatigues in the three time points, the question (severity of fatigue in the current) was adopted with a Likert-type scale from 1 (mild fatigue) to 10 (severe fatigue). 

### 2.2. Cytokine Markers and Biochemistry Assessments

The cytokines evaluated included serum interferon-gamma (IFN-gamma), interleukin (IL)-1, IL-2, IL-5, IL-6, IL-12 p70, tumor necrosis factor-alpha (TNF-alpha), and granulocyte-macrophage colony-stimulating factor (GM-CSF). In addition, biochemistry profiles included the serum white blood cell (WBC) counts, C-reactive protein (CRP), albumin, and prealbumin. These aforementioned tests were performed on POD 0, POD 1, and POD 7. We obtained early morning, fasted venous blood samples from each patient under aseptic techniques. Serum was isolated via centrifugation at 1000× *g* at 20 °C. Subsequent collection of the serum fraction was performed by using standard serum separator tubes, which were then stored at −80 °C until further use for analysis. Serum levels of IFN-gamma, IL-1, IL-2, IL-5, IL-6, IL-12 p70, TNF-alpha, and GM-CSF were determined by using ProcartaPlex multiplex immunoassays panels, affymetrix (eBioscience, EPX110-10810-901), and Luminex 100/200. Furthermore, serum levels of IL-10 cytokine were analyzed via the ELISA kit (Human Platinum ELISA, eBioscience, an affymetrix) according to the manufacturer’s instructions. 

### 2.3. Medical and Demographic Data

Clinicopathologic and medical data were obtained by reviewing the medical charts of patients. We collected the following data on the comorbidity of patients before gastrectomy by using the International Classification of Diseases, Ninth Revision, Clinical Modification (ICD-9-CM codes): anemia (ICD-9-CM: 285.x), myocardial infarction (410.x and 412.x), mild liver disease (571.2 and 571.4–571.6), hyperlipidemia (272.0–272.2), diabetes mellitus (250.0–250.3, 250.7), chronic obstructive pulmonary disease (COPD; 490.x–496.x), renal failure (584.x–586.x), and hypertension (401.x–405.x). Next, the Charlson comorbidity index (CCI) scores were calculated to determine the baseline comorbidity of each patient [29]. Further, the Malnutrition Universal Screening Tool was used to screen the malnourished patients, and overall risk for malnutrition was established as low (score = 0), medium (score = 1), or high risk (score > 2) [30]. To determine the oncological variables, including cancer histology and cancer stage, the American Joint Committee on Cancer (AJCC) staging (8th edition) was implemented [31].

### 2.4. Statistical Analysis

In this study, all statistical analyses were performed using STATA version 15 (Stata Corp LP, College Station, TX, USA). Data are presented as mean (standard deviation), number (percentage), or coefficients and 95% confidence interval (CI). We compared categorical variables using the χ^2^ test or Fisher’s exact test if the numbers were <5. Continuous data were compared using the nonparametric Mann–Whitney *U* test, and some continuous variables were dichotomized according to predetermined thresholds, which were analyzed as categorical variables. The comparison among the three time-points was performed using the *ANOVA* test. In addition, we performed linear regression analysis to assess the correlation between changes in fatigue scores and the changes in the cytokine levels after adjusting for clinicopathologic characteristics, CCI scores, and cancer staging. The independent variable was the change in fatigue scores among time points, and the dependent variables were identified according to the bivariate correlations. All statistics were 2-tailed, and differences were considered statistically significant at *P* < 0.05.

## 3. Results

In this study, we enrolled 34 GA in the analysis (Table 1), including 12 men and 22 women. The mean age was 68.9 years. About the malnutrition risk, the numbers in low, medium, and high risk were 13 (38%), 10 (30%), and 11 (32%), respectively. On determining the AJCC stage, there were 7 (21%) stage I, 3 (9%) stage II, 13 (38%) stage III, and 11 (32%) stage IV tumors. In addition, 4 (12%) cases had a CCI score >3. 

### 3.1. Perioperative Changes in Fatigue Scores

**Figure 1:**
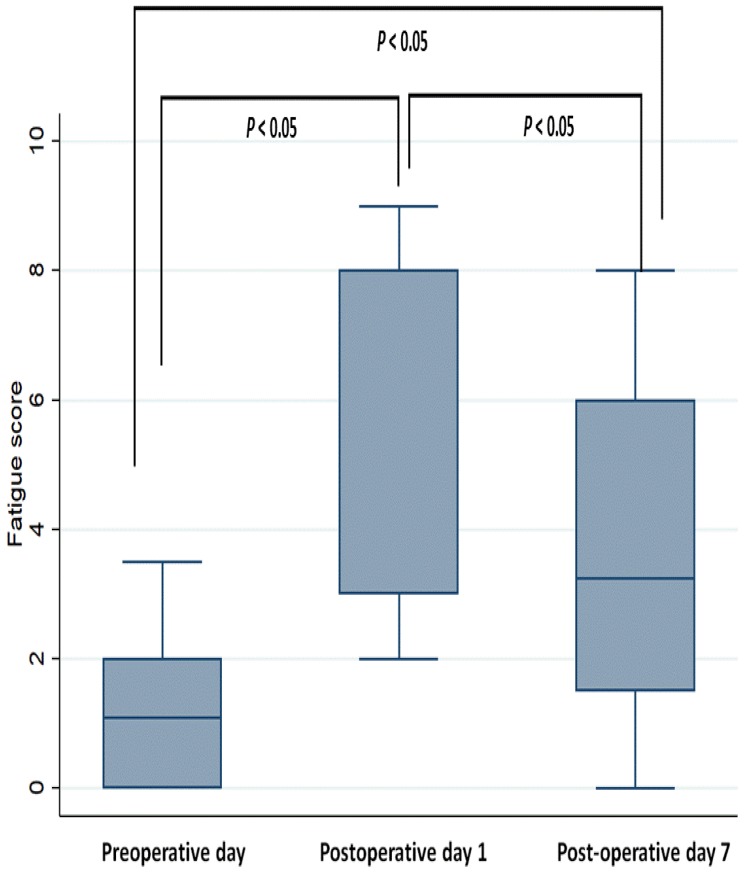
Perioperative fatigue scores.

The mean score of fatigue on POD 0, POD 1, and POD 7 was 1.7, 6.2, and 3.6, respectively. The fatigue score was significantly higher on POD 1 and POD 7 than on POD 0 (both *P* < 0.001; Figure 1). Furthermore, the score significantly decreased on POD 7 compared to that on POD 1 (*P* < 0.001). 

### 3.2. Perioperative Changes in Cytokines Markers and Biochemistry Data

**Figure 2:**
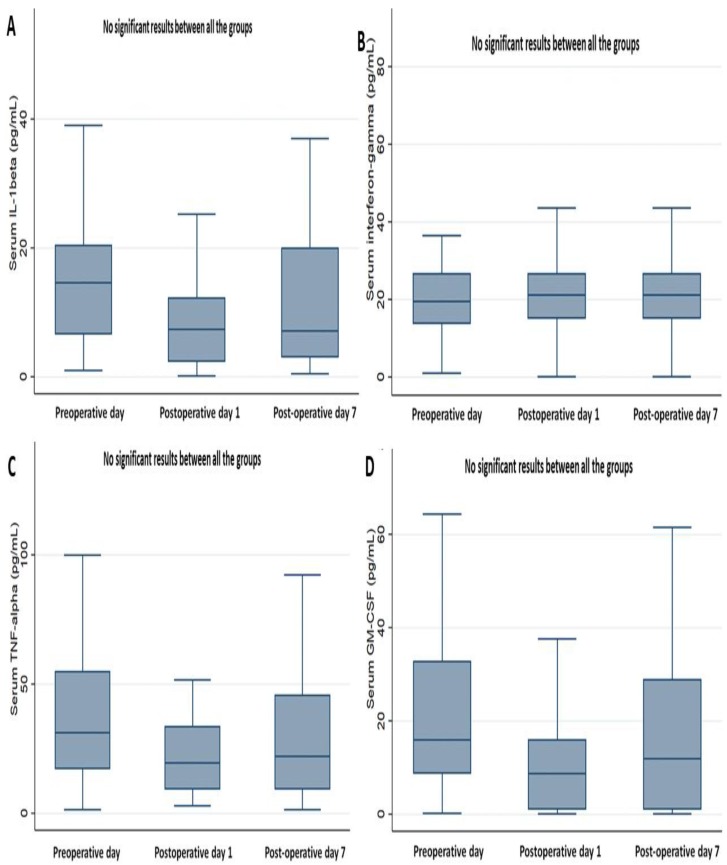
Perioperative change of serum cytokines, part 1. (**A**) Interleukin (IL)-1 beta, (**B**) Interferon-gamma, (**C**) TNF (tumor necrosis factor)-alpha, (**D**) Granulocyte-macrophage colony-stimulating factor (GM-CSF).

**Figure 3:**
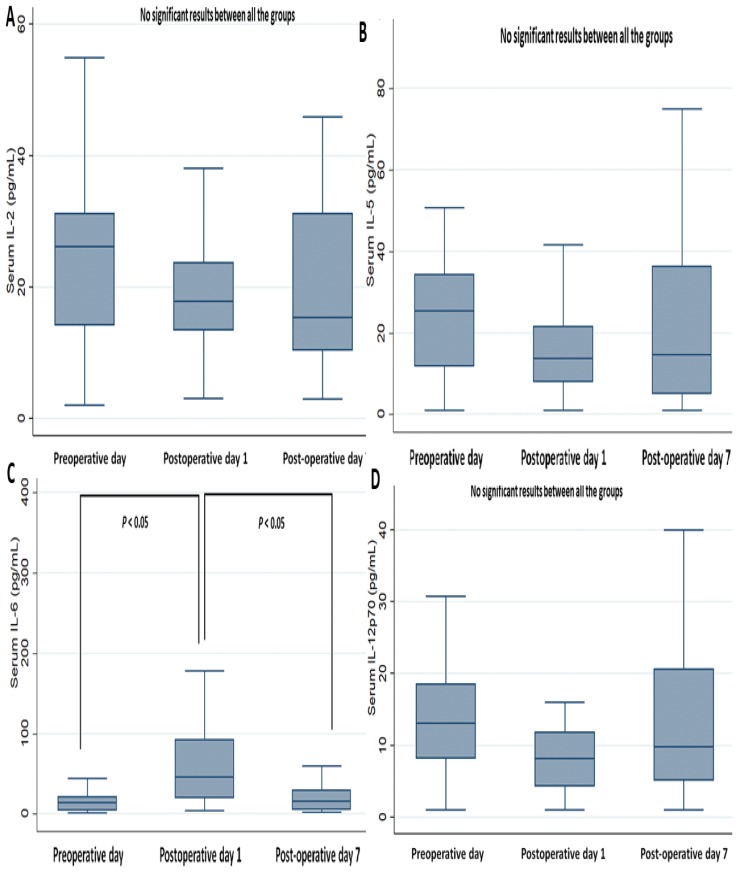
Perioperative change of serum cytokines, part 2. (**A**) IL-2, (**B**) IL-5, (**C**) IL-6, (**D**) IL-12p70.

On determining the perioperative changes in cytokines (Figure 2 and Figure 3), most cytokines (IFN-gamma, IL-1, IL-2, IL-5, IL-12 p70, TNF-alpha, and GM-CSF) showed no significant perioperative change, except for the serum level of IL-6. The mean level of IL-6 significantly increased on POD 1, more than on POD 0 and POD 7 (both *P* < 0.001), while there were no significant differences in the levels between POD 0 and POD 7 (Figure 3C).

**Figure 4:**
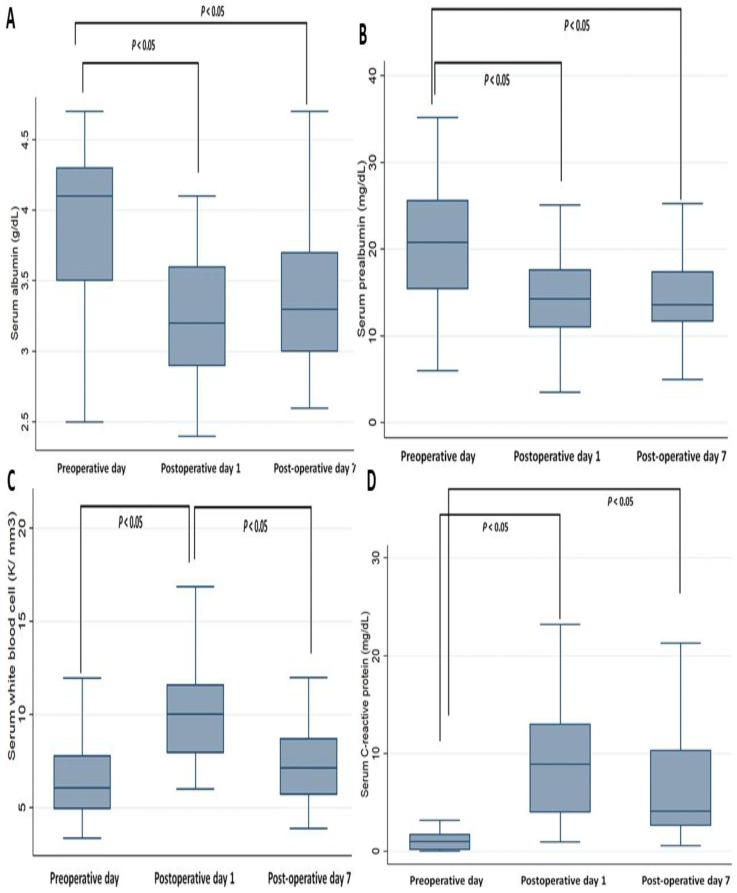
Perioperative change of serum biochemistry profiles. (**A**) Albumin, (**B**) prealbumin, (**C**) white blood cell count, (**D**) C-reactive protein.

Among the biochemistry profiles (Table S1 and Figure 4), the levels of serum albumin and prealbumin on POD 1 and POD 7 significantly decreased compared to those on POD 0; there were no significant differences between the levels on POD 0 and POD 7. The WBC counts significantly increased on POD 1 compared to those on POD 0 and POD 7; there were no significant differences between the levels on POD 0 and POD 7. The CRP levels significantly increased on POD 1 and POD 7 compared to that on POD 0; there were no significant differences between the levels on POD 1 and POD 7. 

### 3.3. Association between Cytokines, Clinicodemographics, Biochemistry Profiles, and Fatigue

**Figure 5:**
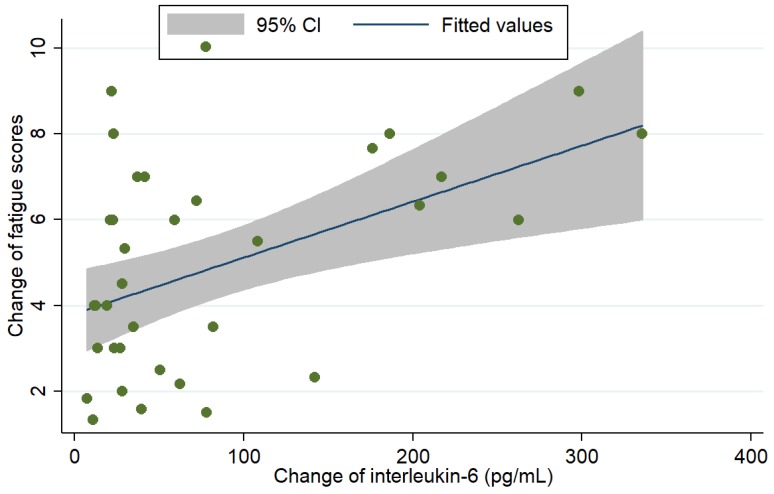
The correlation between the perioperative change in IL-6 and change in fatigue scores (X-axis: perioperative change in IL-6; Y-axis: perioperative change in fatigue scores; *P* value = 0.002, R^2^ = 0.250).

**Figure 6:**
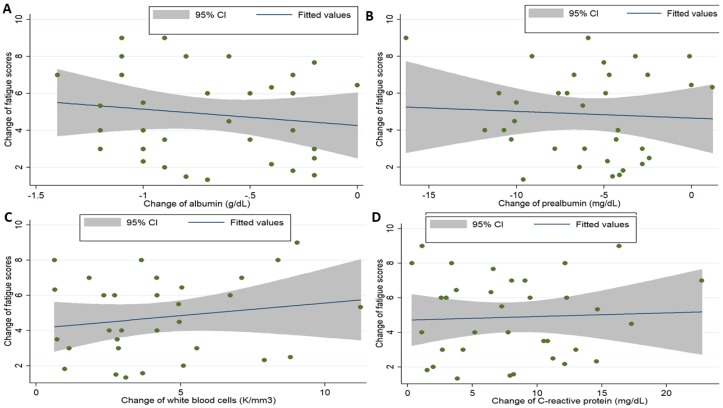
The correlation between the perioperative change in biochemistry profiles and change in fatigue scores (X-axis: perioperative change in biochemistry profiles; Y-axis: perioperative change in fatigue scores; albumin: *P* value = 0.427, R^2^ = 0.019; prealbumin: *P* value = 0.744, R^2^ = 0.003; white blood cells: *P* value = 0.654, R^2^ = 0.006; C-reactive protein: *P* value = 0.789, R^2^ = 0.002).

Among the cytokines, only IL-6 was included as a dependent variable to predict the association between the change in fatigue scores from POD 0 to POD 1. The scatter plot graph illustrated the association between perioperative changes in IL-6 and fatigue scores (Figure 5; *P* value = 0.002, R = 0.250) and between the changes in biochemistry profiles and fatigue scores (Figure 6). It showed that change of IL-6 had a strong correlation with the change of fatigue scores. Furthermore, logistic regression analysis was performed after adjusting age, gender, malnutrition risk, AJCC stages, and change in IL-6 levels (Table 2). The analysis showed that changes in IL-6 levels (coefficients = 0.01 for every 1 pg/mL increment; 95% confidence interval: 0.01–0.02; *P* = 0.037) and high malnutrition risk (coefficients = 2.80; 95% confidence interval: 1.45–3.52; *P* = 0.041) were positively significantly associated with increased scores for perioperative fatigue. 

## 4. Discussion

The results of our study showed that gastric surgery contributed to perioperative fatigue in GA patients. During the perioperative period, the highest score of fatigue occurred on POD 1, but patients partially recovered on POD 7 compared to the preoperative baseline. Considering the perioperative change in cytokine levels, IL-6 significantly increased on POD 1 compared to POD 0, which was associated with the severity of fatigue on adjusted analysis.

IL-6, a pleiotropic cytokine involved in both wound healing and regeneration in mitotic tissues [32], is a pro-inflammatory molecule and may have certain anti-inflammatory properties [33]. IL-6 works via the IL-6/membrane IL-6R/glycoprotein 130 pathway to activate JAK/STAT-3 and ERK cascades associated with inflammatory and mitotic processes, respectively [34,35]. Notably, the clinical study showed that the advanced cancer patients with elevated levels of plasma IL-6 simultaneously developed malnutrition and were associated with anemia, anorexia, and depression [33,36]. Furthermore, chronic IL-6 exposure in cancer patients may induce not only wasting in skeletal muscles due to increased protein breakdown and decreased induction of post-prandial muscle protein synthesis [37], but also tumorigenesis [38]. 

In addition to the production of IL-6 by muscles or osteoblasts, several types of cancer and associated stromal cells are involved in the production of this cytokine [39], which causes tumor growth and invasiveness in terminal cancer patients [40]. Notably, gastric cancer was associated with elevated IL-6 levels in biological fluids [41,42]. In this study, the preoperative level of IL-6 had increased in more than 30% of the patients, which also validated the aforementioned phenomenon that gastric cancer or the cancer microenvironment could produce IL-6. There are several reasons to explain why the level of IL-6 in plasma significantly increased on POD 1, including surgical stress or physical trauma that induced the production of IL-6 from normal human cells—as IL-6 is an essential factor for wound healing [43] —and from the cancer microenvironment. Furthermore, this elevation in the IL-6 level that was detectable in plasma indicates the role of IL-6 in cancer biology, as well as the clinical implication of IL-6 as a biomarker to detect fatigue or follow the responses to therapy. 

As IL-6 has emerged as a cytokine causing fatigue in GA patients, therapeutic strategies focusing on IL-6 and associated signaling could be used to attenuate fatigue [44]. In animal models, the inhibition of the IL-6 signaling cascade attenuated cachexia progression [12] and muscle loss [15]. In early clinical studies, some drugs have shown promising results by attenuating the symptoms of cancer cachexia even though they do not directly target the IL-6 pathway [45,46]. The humanized IL-6Rab, tocilizumab, reduced plasma IL-6 levels, attenuated muscle loss, and restored levels of plasma albumin in lung cancer patients [16,47]. In addition, a recent double-blinded randomized clinical trial demonstrated that astragalus-based herbal medicine could effectively reduce cancer-related fatigue in advanced cancer patients (i.e., lung, colon, and breast cancer) [48]. However, further studies are required to validate their effects in GA patients undergoing major operations. 

In our study, malnutrition was the other risk associated with perioperative fatigue, and one recent study focusing on colectomy patients had similar finding [49]. To our knowledge, malnutrition is a common presentation in GA patients due to cancer cachexia and the obstruction of gastrointestinal tract. For the surgical patients, the increased physical metabolism and insufficient calorie intake further deteriorate the metabolism balance, which causes minor (fatigue or delayed recovery) or major adverse effects (complication or mortality) [50]. Therefore, preoperative screening is very imperative to recognize the malnourished patients, who need nutrition intervention program [51]. In selected cases, deferring the operation may be considered to prevent aforementioned complications. 

Another explanation for the relationship between elevated IL-6 and fatigue may be related to the anxiety in cancer patients. Anxiety is another common mental illness in cancer patients, who often worry about the cancer stage or oncological outcomes. Studies showed that the patient with chronic anxiety were associated with elevated serum IL-6 values, which caused further neurobehavioral complications [52]. As a result, IL-6 could deteriorate the mental and physical functions in cancer patients in a vicious cycle. Further studies should investigate this association, and surgical staff could adopt an anxiety-relieving program for cancer patients. 

The findings of our study should be evaluated in the context of some limitations. First, the study is limited to an Asian population, and further studies are warranted to validate these findings in Western populations. Second, the impact of the changes of IL-6 serum values on fatigue in the late postoperative period should also be investigated in the future. 

## 5. Conclusions

More than 30% of GA patients had elevated baseline levels of plasma IL-6 and the levels of plasma IL-6 were associated with the worsening of perioperative fatigue in GA patients. Nevertheless, further clinical studies are needed to validate whether targeting the IL-6 signaling cascade or new fatigue-targeting medications may attenuate fatigue in GA patients undergoing surgical procedures.

## Figures and Tables

**Table 1:** Clinicopathologic characteristics of study subjects (N = 34).

Characteristic	Value
Age, mean (SD)	68.9 (9.7)
Gender	
Male	22 (65%)
Female	12 (35%)
Charlson comorbidity index scores	
≤3	30 (88%)
>3	4 (12%)
Malnutrition Universal Screening Tool	
Low risk	13 (38%)
Medium risk	10 (30%)
High risk	11 (32%)
The American Joint Committee on Cancer stage	
I	7 (21%)
II	3 (9%)
III	13 (38%)
IV	11 (32%)

**Table 2:** Adjusted linear regression analysis for predicting the increase in fatigue scores between preoperative day 0 and postoperative day 1.

	Coefficients	95% Confidence Interval	*P*-Value
Change in IL-6 levels	0.01	(0.01, 0.02)	0.037
Age	0.03	(−0.06, 0.11)	0.470
Male gender (ref: female)	0.33	(−1.30, 1.98)	0.678
Malnutrition risk * (ref: low risk)			
Medium risk	1.42	(−0.12, 1.56)	0.785
High risk	2.80	(1.45, 3.52)	0.041
AJCC stage (ref: stage I)			
II	2.58	(−0.87, 6.04)	0.137
III	0.97	(−1.32, 3.27)	0.392
IV	1.72	(−0.57, 4.02)	0.135

Note: IL: interleukin; ref: reference; AJCC: the American Joint Committee on Cancer; *: Malnutrition Universal Screening Tool.

## Supplementary Materials

The following are available online at https://www.mdpi.com/2077-0383/8/4/543/s1, Table S1: Cytokine levels, biochemistry profiles, and fatigue scores on preoperative day 0, postoperative day 1, and postoperative day 7 (*N* = 34).
