# Supplementary material for: Association between Interleukin-6 Levels and Perioperative Fatigue in Gastric Adenocarcinoma Patients
Source: J Clin Med. 2019 Apr 20;8(4):543. doi: 10.3390/jcm8040543 (PMC6518263; doi:10.3390/jcm8040543)
Supplement: Supplementary file 1 [file jcm-08-00543-s001.pdf]

Table S1. Cytokine levels, biochemistry profiles, and fatigue scores on preoperative day 0, postoperative day 1, and postoperative day 7 (N = 34).

Factor, mean (SD)	Preoperative day 0	Postoperative day 1	Postoperative day 7	*P*-value
IL-1 beta (pg/mL)	16.0 (12.6)	10.1 (10.9)	12.2 (11.6)	N.S.
IL-2 (pg/mL)	25.6 (12.8)	19.7 (12.1)	20.6 (12.9)	N.S.
IL-5 (pg/mL)	25.9 (20.0)	18.7 (19.5)	20.6 (18.1)	N.S.
IL-6 (pg/mL)	17.6 (16.7)	86.5 (104.7)	19.8 (15.1)	#, *
IL-12p70 (pg/mL)	15.6 (10.3)	10.9 (10.2)	13.5 (10.9)	N.S.
Interferon-gamma (pg/mL)	22.4 (17.2)	22.0 (10.6)	22.0 (10.6)	N.S.
TNF-alpha (pg/mL)	40.3 (32.1)	28.5 (32.7)	30.2 (27.0)	N.S.
GM-CSF (pg/mL)	22.8 (20.0)	13.3 (17.2)	17.8 (18.7)	N.S.
White blood cells (K/mm^3)	6.7 (2.7)	10.4 (3.5)	7.7 (2.8)	#, *
C-reactive protein (mg/dL)	1.7 (2.6)	9.6 (6.5)	6.3 (5.3)	#, +
Albumin (g/dL)	3.9 (0.5)	3.3 (0.4)	3.4 (0.5)	#, +
Prealbumin (mg/dL)	21.0 (7.5)	14.4 (5.0)	14.4 (5.0)	#, +
Fatigue, mean (SD)	1.7 (2.3)	6.2 (2.8)	3.6 (2.6)	#, *, +

Note: N.S. *P*-value > 0.05 in any comparisons

P-value < 0.05 if comparison was made between preoperative day 0 and postoperative day 1; * *P*-value < 0.05 if comparison was made between postoperative day 1 and postoperative day 7; + *P*-value < 0.05 if comparison was made between preoperative day 0 and postoperative day 7.
